# Research on Lipidomic Profiling and Biomarker Identification for Osteonecrosis of the Femoral Head

**DOI:** 10.3390/biomedicines12122827

**Published:** 2024-12-12

**Authors:** Yuzhu Yan, Jihan Wang, Yangyang Wang, Wenjing Wu, Wei Chen

**Affiliations:** 1 Department of Laboratory Medicine, The First Affiliated Hospital of Xi’an Jiaotong University, Xi’an 710061, China; 2 Clinical Laboratory of Honghui Hospital, Xi’an Jiaotong University, Xi’an 710054, China; 3 Institute of Medical Research, Northwestern Polytechnical University, Xi’an 710072, China; 4 School of Electronics and Information, Northwestern Polytechnical University, Xi’an 710129, China

**Keywords:** osteonecrosis of the femoral head, lipidomic profile, feature selection, LASSO, diagnostic biomarker

## Abstract

**Objectives:** Abnormal lipid metabolism is increasingly recognized as a contributing factor to the development of osteonecrosis of the femoral head (ONFH). This study aimed to explore the lipidomic profiles of ONFH patients, focusing on distinguishing between traumatic ONFH (TONFH) and non-traumatic ONFH (NONFH) subtypes and identifying potential biomarkers for diagnosis and understanding pathogenesis. **Methods:** Plasma samples were collected from 92 ONFH patients (divided into TONFH and NONFH subtypes) and 33 healthy normal control (NC) participants. Lipidomic profiling was performed using ultra-high performance liquid chromatography–tandem mass spectrometry (UHPLC–MS/MS). Data analysis incorporated a machine learning-based feature selection method, least absolute shrinkage and selection operator (LASSO) regression, to identify significant lipid biomarkers. **Results:** Distinct lipidomic signatures were observed in both TONFH and NONFH groups compared to the NC group. LASSO regression identified 11 common lipid biomarkers that signify shared metabolic disruptions in both ONFH subtypes, several of which exhibited strong diagnostic performance with areas under the curve (AUCs) > 0.7. Additionally, subtype-specific lipid markers unique to TONFH and NONFH were identified, providing insights into the differential pathophysiological mechanisms underlying these subtypes. **Conclusions:** This study highlights the importance of lipidomic profiling in understanding ONFH-associated metabolic disorders and demonstrates the utility of machine learning approaches, such as LASSO regression, in high-dimensional data analysis. These findings not only improve disease characterization but also facilitate the discovery of diagnostic and mechanistic biomarkers, paving the way for more personalized therapeutic strategies in ONFH.

## 1. Introduction

Osteonecrosis of the femoral head (ONFH) is characterized by the death of bone cells in the femoral head. ONFH primarily results from a compromised blood supply and subsequently leads to joint collapse, severe pain, and functional impairment [1,2]. It can be broadly classified into two main categories: traumatic ONFH (TONFH) and non-traumatic ONFH (NONFH) [3,4,5]. TONFH typically occurs from direct injuries, such as fractures or dislocations, which disrupt the blood flow to the femoral head [6,7]. NONFH arises from various etiological factors, including prolonged corticosteroid use, excessive alcohol consumption, hypercoagulable states, hematologic disorders, metabolic diseases, etc. [8,9]. Discriminating between TONFH and NONFH is essential as it dictates different therapeutic approaches and prognoses, ensuring optimized patient management and outcomes.

An abnormal lipid metabolism has been considered one of the etiological factors for ONFH [10,11,12]. Disorders in lipid homeostasis can lead to the accumulation of fat cells in bone marrow, which may compress blood vessels, inhibiting blood flow to the femoral head and leading to bone cell death [13,14,15]. Additionally, conditions like hyperlipidemia are often observed in ONFH patients, which can result in the formation of lipid emboli and further obstruction of the vascular supply [16,17]. Chronic exposure to certain risk factors, such as excessive alcohol consumption or prolonged corticosteroid use, can exacerbate these lipid metabolic abnormalities, thereby increasing the risk of ONFH [18,19]. However, the specific lipidomic alterations that underlie ONFH remain poorly understood, especially with regard to how these changes differ between traumatic and non-traumatic forms of the disease. Understanding these lipidomic profiles could reveal novel biomarkers and therapeutic targets, ultimately leading to improved management strategies.

Nowadays, metabolome studies have emerged as a promising field for research on metabolic diseases [20,21,22]. The metabolome encompasses the complete set of small-molecule metabolites present within an organism, tissue, or cell. The lipidome, a specialized subset of the metabolome, focuses on the comprehensive profiling of lipids, which play pivotal roles in various biological processes, including signaling, energy storage, insulation, etc. [23,24,25,26]. By exploring the lipidomic landscape of ONFH, researchers can unravel the potential mechanistic pathways and identify the specific lipid biomarkers of the disease [27]. It is worth noting that metabolomic and lipidomic studies often yield vast and complex datasets. These high-dimensional datasets pose significant challenges in terms of analysis and interpretation [28,29]. Machine learning, a subset of artificial intelligence, has demonstrated profound applications in the analysis of complex biological datasets, such as those generated by metabolomic or lipidomic studies [30,31,32]. Feature selection methods, a specialized domain within machine learning, are designed to identify and retain the most informative features of a dataset while discarding the redundant or irrelevant ones [33,34]. This not only enhances the efficiency and accuracy of the subsequent analyses, but also helps to reduce the computational burden. In the context of metabolomic or lipidomic research, feature selection can identify the key metabolites or lipids that are most indicative of a particular condition or disease [35,36].

The aim of this study was to identify and characterize the lipidomic alterations in both the TONFH and NONFH subtypes compared to the normal controls. We hypothesized that the lipidomic profiles differ significantly between these subtypes and that specific lipid biomarkers may be associated with disease onset and progression. By using ultra-high performance liquid chromatography–tandem mass spectrometry (UHPLC–MS/MS), we conducted a comprehensive analysis of the blood samples from three distinct groups: TONFH, NONFH, and normal controls (NCs). Additionally, we applied feature selection methods to isolate the key lipid features unique to each group. This research aimed to uncover specific lipid biomarkers that could serve as diagnostic tools and therapeutic targets for ONFH, providing new insights into the disease’s underlying pathophysiological mechanisms.

## 2. Materials and Methods

### 2.1. Participant Information

This retrospective cohort study enrolled 92 patients with ONFH and 33 NC participants who were treated or underwent physical examinations at Honghui Hospital, Xi’an Jiaotong University. The samples were collected during the period from 2020 to 2022. The ONFH patients were recruited according to the updated version of the Association Research Circulation Osseous (ARCO) staging system from 2019, ensuring that precise diagnostic criteria were met. Specifically, ONFH was confirmed through a combination of clinical symptoms, imaging findings, and the ARCO classification system. The 92 ONFH patients were further classified into two subtypes—46 cases of traumatic ONFH (TONFH) and 46 cases of non-traumatic ONFH (NONFH)—based on a detailed patient history and etiology. To address potential recall bias, a detailed medical review of the patients’ records and imaging was conducted to corroborate self-reported trauma history. In cases where the trauma history was unclear or inconsistent, clinical documentation and imaging evidence were used as the primary criteria for classification. Patients with a history of fragility fractures, as well as those using bone resorption inhibitors or antihyperlipidemic drugs, were excluded to avoid potential confounding effects on lipid metabolism. The NC participants were defined as individuals with no clinical history or radiographic evidence of ONFH or other significant bone-related diseases. To minimize potential bias, stringent inclusion criteria were applied to the NC group. Individuals with any comorbidities known to affect lipid metabolism, such as osteoporosis (OP), hypertension, hyperlipidemia, gout, or hyperuricemia, were excluded to ensure the NC group was as representative of a healthy population as possible, without confounding factors that could influence the lipidomic profiles. This study was approved by the Institutional Review Board of Honghui Hospital, Xi’an Jiaotong University (No. 202005002), and registered in the Chinese Clinical Trial Registry (ChiCTR) (Registration No.: ChiCTR2000033442).

### 2.2. Blood Sample Collection and Lipid Extraction

Plasma samples were collected for lipidome detection. The participants were to avoid exercise and alcohol within 24 h before blood collection, and maintain a normal diet and get adequate sleep. Fasting venous blood was collected early the next morning.

Methanol (0.75 mL) was added to a 100 μL sample, which was placed into a glass tube with a Teflon-lined cap, and the tube was vortexed. An amount of 2.5 mL of MTBE was added and the mixture was incubated for 1 h at room temperature in a shaker. Phase separation was induced by adding 0.625 mL of MS-grade water. After 10 min of incubation at room temperature, the sample was centrifuged at 1000× *g* for 10 min. The upper (organic) phase was collected, and the lower phase was re-extracted with 1 mL of the solvent mixture, and the upper phase was collected. The combined organic phases were dried and dissolved in 100 μL of isopropanol for storage.

### 2.3. UHPLC-MS/MS Analysis

The UHPLC-MS/MS analyses were performed using a Vanquish UHPLC system (Thermo Fisher, Bremen, Germany) coupled with an Orbitrap Q ExactiveTM HF mass spectrometer (Thermo Fisher, Bremen, Germany) by Novogene Co., Ltd. (Beijing, China). The primary focus of this analysis was achieving the high-resolution separation of the lipid species, with particular emphasis on isomeric separation.

### 2.4. Data Search

The raw data files generated by the UHPLC-MS/MS were processed using Compound Discoverer 3.01 (CD3.1, Thermo Fisher, Waltham, MA, USA) to perform peak alignment, peak picking, and quantification for each metabolite. After that, the peak intensities were normalized to the total spectral intensity. The normalized data were used to predict the molecular formula based on additive ions, molecular ion peaks, and fragment ions. And then, the peaks were matched with LIPID MAPS [37] and LipidBlast [38] to obtain accurate qualitative and relative quantitative results.

### 2.5. Feature Selection Methods

Given the high-dimensional nature of lipidomic datasets, where the number of features often far exceeds the number of samples, traditional regression models can overfit and lead to a poor predictive performance. Herein, we performed a least absolute shrinkage and selection operator (LASSO) regression [39,40] to identify the key lipid metabolites associated with TONFH and NONFH, respectively. Choosing a LASSO regression as the feature selection method offered several advantages over other techniques. Firstly, LASSO performs both feature selection and regularization simultaneously, allowing for the identification of the most relevant features while shrinking the coefficients of the less important ones. This helps to avoid overfitting and improve the generalization of the model. Additionally, LASSO can handle multicollinearity effectively by selecting only one feature from a group of highly correlated features. The model of Lasso regression is as follows. In the model, y is the dependent variable, $x_{i}\in X$ is the independent variable, ω is the regression coefficient, λ is the regularization parameter, and $\lambda {\left|\left|\omega \right|\right|}_{1}$ represents the L1 normal form of λ. In the LASSO regression, a 10-fold cross-validation model is used to increase the robustness of the model results.

$$w=argmin\overset{N}{\underset{i=1}{\sum }}{\left(y_{i}-w^{T}x_{i}\right)}^{2}+\lambda {\left|\left|w\right|\right|}_{1}$$

### 2.6. Statistical Analysis

The statistical analysis in this study was mainly performed using R version 4.0.2. A chi-square test and Wilcoxon rank-sum test were used to test the statistical significance of the different groups in terms of the basic clinical information (*p* < 0.05 was considered as a significant difference). A Pearson correlation analysis was performed to evaluate the correlation among the QC samples based on the lipidomic profiling. A partial least squares-discrimination analysis (PLS-DA), t-distributed stochastic neighbor embedding (t-SNE), and receiver operating characteristic (ROC) analysis were performed using the “mixOmics”, “Rtsne”, and “pROC” packages in R, respectively. The area under the curve (AUC) values were obtained to evaluate the performance of the candidate lipids as disease diagnostic markers.

## 3. Results

### 3.1. Basic Clinical Information on the Participants

We collected a total of 125 plasma samples for 46 cases of TONFH, 46 cases of NONFH, and from 33 NC subjects for lipidomic detection. The detailed clinical demographics of the participants are presented in Table 1. The results of the chi-square tests and Wilcoxon rank-sum tests revealed no significant differences across the three groups with respect to age distribution, gender composition, or Body Mass Index (BMI) values, nor for the ARCO staging composition between the TONFH and NONFH groups, indicating a well-matched cohort that mitigates the confounding demographic variables in the subsequent lipidomic analysis.

### 3.2. Overview of Lipidomic Profiling in TONFH, NONFH, and NC Samples

**Figure 1:**
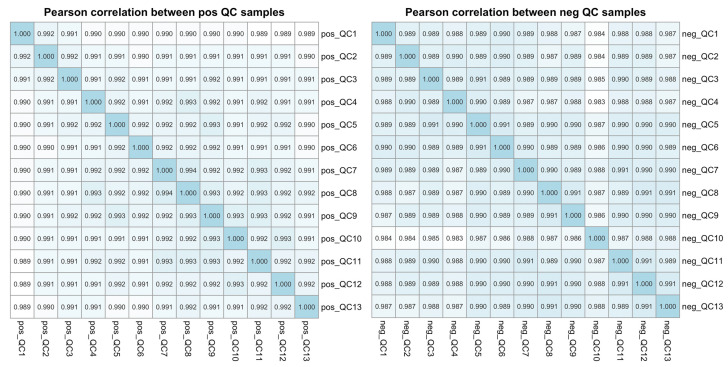
The Pearson correlation analysis among the QC samples based on the LC-MS/MS lipidomic profiling data. The plot illustrates the Pearson correlation coefficients for each pair of quality control (QC) samples in both the positive (pos) and negative (neg) ion modes, providing a measure of the reproducibility and reliability of the lipidomic data across the QC samples.

**Figure 2:**
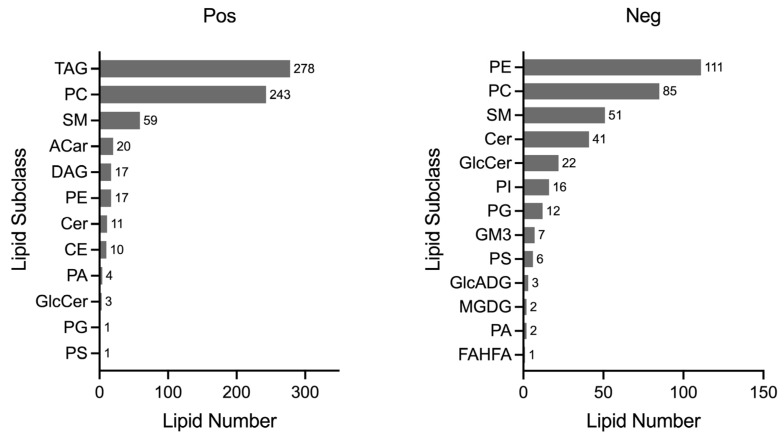
Lipid subclass analysis. A total of 664 lipid compounds were annotated in the positive mode, and 359 lipid compounds in the negative mode, each classified into their respective subclasses. The horizontal axis quantifies the number of lipid compounds, while the vertical axis lists the names of each lipid subclass. TAG: triacylglyceride; PC: phosphatidylcholine; SM: sphingomyelin; ACar: acylcarnitine; DAG: diacylglycerol; PE: phosphatidylethanolamines; Cer: ceramide; CE: cholesteryl ester; PA: phosphatidic acid; GlcCer: glucosylceramide; PG: phosphatidylglycerol; PS: phosphatidylserine; PI: phosphatidylinositol; GM3: ganglioside; GlcADG: glycosyldiacylglycerols; MGDG: monogalactosyldiacylglycerol; FAHFA: fatty acid esters of hydroxy fatty acids.

All 125 plasma samples were subjected to lipidome profiling detection utilizing UHPLC-MS/MS high-throughput technology, operating in both the positive and negative ion modes. To ensure the integrity and consistency of the lipidomic data, quality control (QC) samples were prepared by pooling equal aliquots from each study sample. These QC samples were strategically interspersed within the analytical sequence, positioned at the outset and subsequently after every ten sample injections. This rigorous QC protocol yielded a total of 13 QC samples, providing a robust framework for monitoring the performance and reliability of the lipidomic analysis throughout this study. Compound Discoverer software was applied to analyze the LC-MS/MS-based lipidomic data. After a quantitative analysis, our study yielded a comprehensive extraction of lipid compounds, with a total of 852 identified in the positive ion mode and 506 in the negative ion mode across all the samples, inclusive of the QC samples. The Pearson correlation coefficients calculated for the QC samples were impressively close to unity, ranging from 0.987 to 0.994, as shown in Figure 1. Such high correlation values are indicative of exceptional stability within the detection system and attest to the high quality of the data acquired in this study. According to the International Lipid Classification and Nomenclature Committee, lipids are divided into eight primary categories, and further subdivided into various subclasses. Within the positive ion mode, we successfully annotated a total of 664 lipid compounds to their respective subclasses. The subclass distribution was notably dominated by triglycerides (TAG), which constituted 41.87% of the identified lipids, followed by phosphatidylcholines (PC) at 36.60%. In the negative ion mode, of the 359 lipid compounds annotated, the most prevalent subclasses were phosphatidylethanolamines (PE), representing 30.92%; phosphatidylcholines (PC) at 23.68%; sphingomyelins (SM) at 14.21%; and ceramides (Cer), accounting for 11.42%. The subclass composition and their relative abundances are summarized in Figure 2, providing a detailed overview of the lipid subclass distribution in this study.

**Figure 3:**
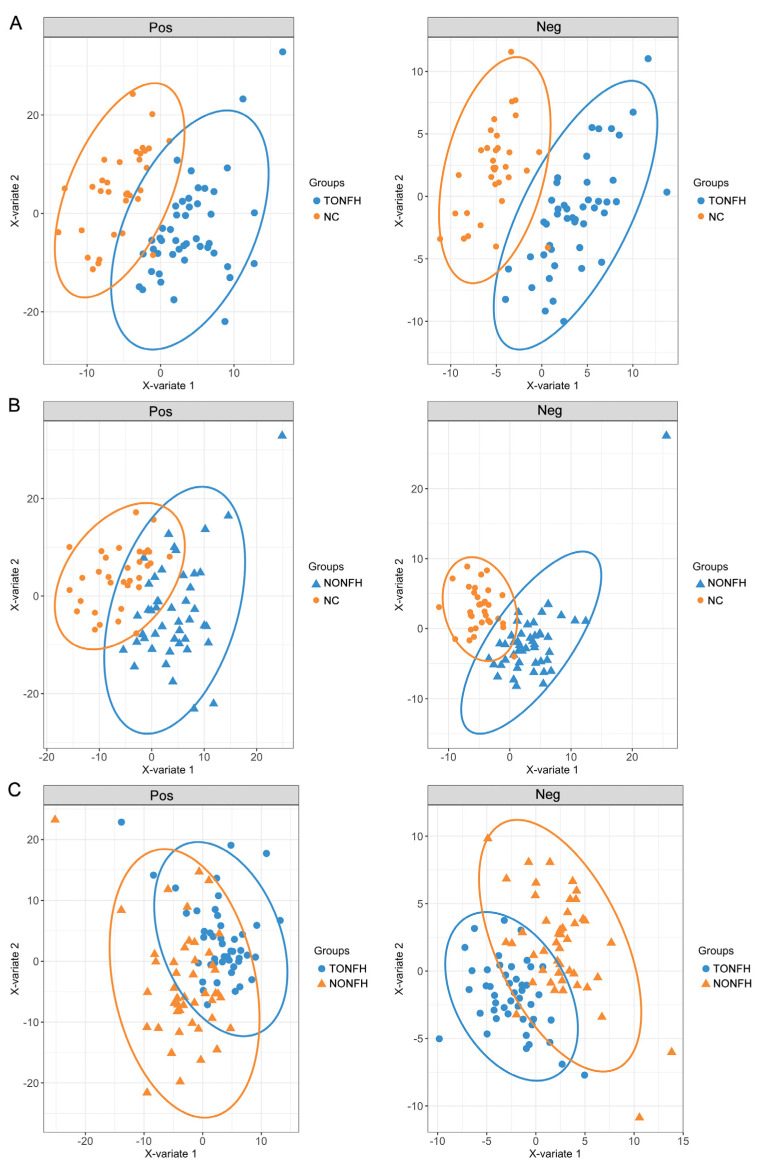
PLS-DA plots based on the lipidomic profiling in the positive (Pos) and negative (Neg) ion modes. The analysis was performed using the “mixOmics” package in the R platform, based on the lipidomic profiling data of the samples. (**A**) PLS-DA plots for TONFH and NC samples; (**B**) PLS-DA plots for NONFH and NC samples; and (**C**) PLS-DA plots for TONFH and NONFH samples.

PLS-DA is one of the most well-known classification procedures in chemometrics. In the lipidomic analysis, we utilized a PLS-DA to elucidate the lipidome signatures to investigate the lipidomic profiles associated with ONFH. The PLS-DA models differentiated TONFH from NC well, as illustrated in Figure 3A, and NONFH from NC, as depicted in Figure 3B. These models revealed distinct lipidomic signatures in each osteonecrosis subtype relative to the normative lipidome, underlying the metabolic alterations inherent to TONFH and NONFH. The discrimination between TONFH and NONFH was less pronounced in the PLS-DA model, with the samples of TONFH and NONFH overlapping to some extent (Figure 3C), suggesting subtle differences in the lipid profiles of these two subtypes of ONFH. The above findings revealed an altered lipidomic signature in the ONFH patients compared to the normal participants, reinforcing the potential of lipidomic analysis for disease characterization and biomarker discovery. The less distinct separation between TONFH and NONFH may imply overlapping lipidomic features or shared metabolic pathways affected in both conditions, despite their different etiologies.

### 3.3. Using LASSO Regression for Lipid Feature Selection

**Figure 4:**
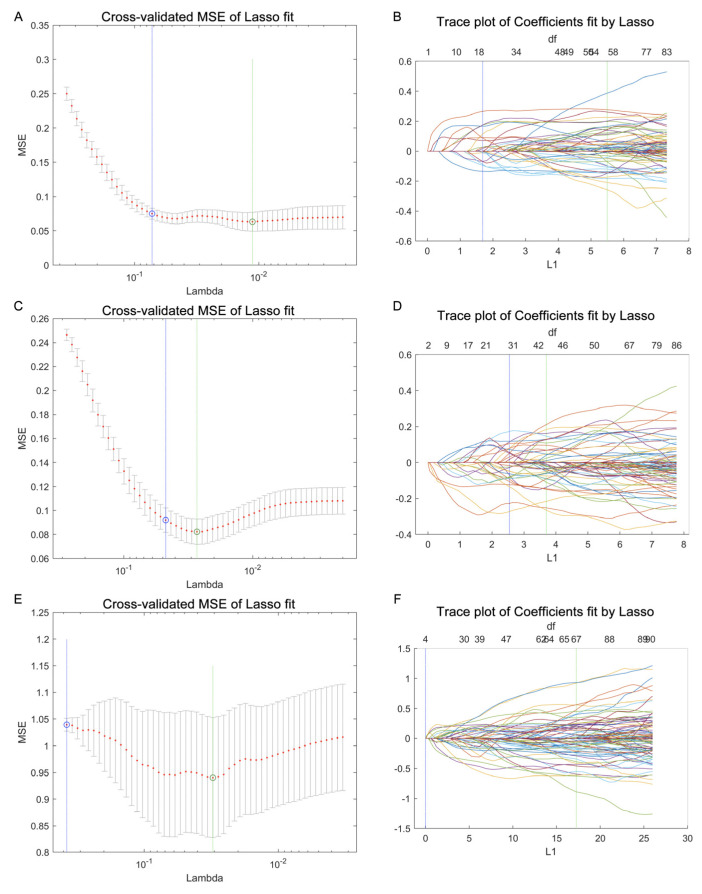
Using a LASSO regression for lipid feature selection across the different groups. The data processing was performed using a LASSO function, and the graph plotting was carried out with the lassoPlot function in MATLAB. The parameter Lambda (λ) was used to determine the optimal set of lipid features. (**A**,**B**) Cross-validated mean squared error (MSE) of LASSO fit and trace plot of coefficients fit by LASSO for TONFH versus NC comparison; (**C**,**D**) cross-validated MSE of LASSO fit and trace plot of coefficients fit by LASSO for NONFH versus NC comparison; and (**E**,**F**) cross-validated MSE of LASSO fit and trace plot of coefficients fit by LASSO for TONFH versus NONFH comparison.

**Figure 5:**
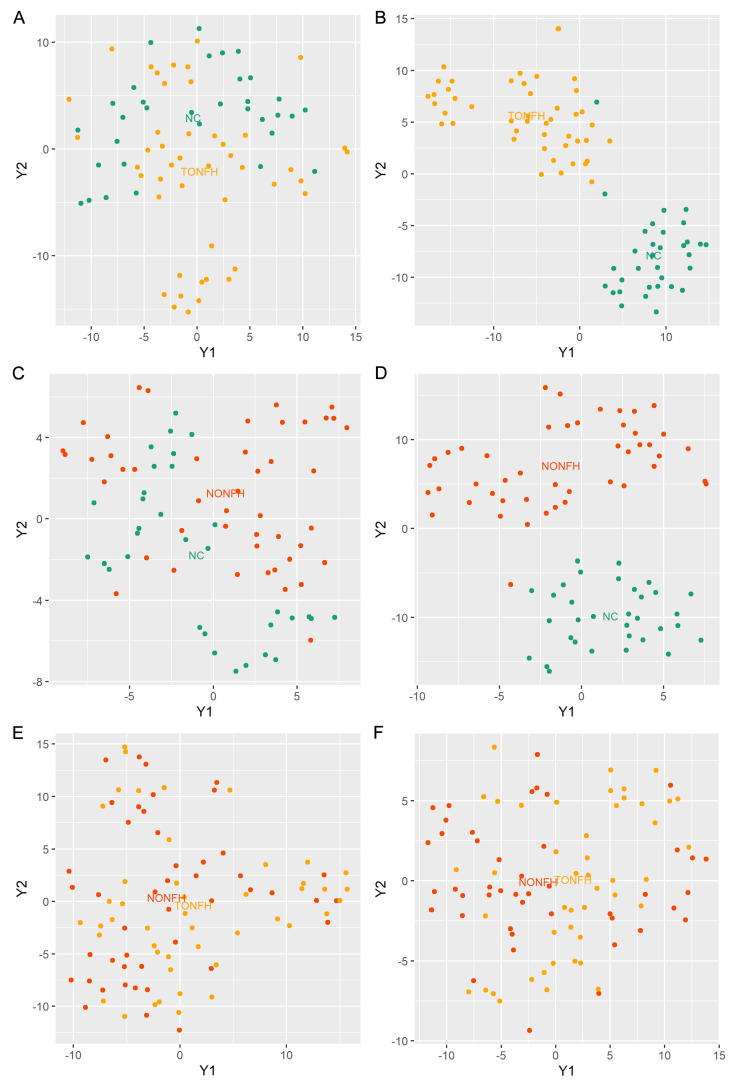
T-SNE plots based on lipidomic profiling. The analysis was performed using the “Rtsne” package in the R platform, based on the lipidomic profiling data of the samples. (**A**) T-SNE plots for TONFH versus NC samples utilizing the entire set of 1358 lipid features; (**B**) T-SNE plots for TONFH versus NC samples utilizing the 56 lipid features selected by LASSO; (**C**) T-SNE plots for NONFH versus NC samples utilizing the entire set of 1358 lipid features; (**D**) T-SNE plots for NONFH versus NC samples utilizing the 43 lipid features selected by LASSO; (**E**) T-SNE plots for NONFH versus NONFH samples utilizing the entire set of 1358 lipid features; and (**F**) T-SNE plots for TONFH versus NONFH samples utilizing the selected 67 lipid features selected by LASSO. Following the LASSO-based feature selection, the discrimination between the groups significantly improved, with a clearer separation of the samples for both the TONFH vs. NC and NONFH vs. NC comparisons.

Utilizing a LASSO regression for selecting lipids from the lipidome data enhances disease discrimination by pinpointing the most predictive lipidomic features while simultaneously reducing model complexity. It applies a shrinkage penalty to the regression coefficients, effectively deleting the less relevant lipids, and retaining the lipidome subset with the most significant contributions to disease classification. Herein, we performed LASSO to obtain the subset of lipids relevant to the classification of TONFH and NONFH. Specifically, in our investigation of the lipidomic signature containing 1358 lipid features (852 lipid compounds in the positive ion model and 506 lipid compounds in the negative ion model), we conducted a comparative analysis across the three cohorts: TONFH, NONFH, and NC. By employing a LASSO regression based on the lipidomic profiling, we identified subsets of lipid features with better discriminative power: 56 lipid features for TONFH versus NC, 43 for NONFH versus NC, and 67 for TONFH versus NONFH classification. The cross-validated mean squared error (MSE) of the LASSO fit and the trace plot of the coefficients fit by LASSO are shown in Figure 4. The application of t-SNE revealed enhanced classification performances after the LASSO feature selection for both TONFH versus NC (Figure 5A,B) and NONFH versus NC (Figure 5C,D), with a particularly notable improvement observed in the NONFH versus NC comparison (Figure 5C,D). However, the differentiation between TONFH and NONFH did not exhibit a significant enhancement after the LASSO feature selection (Figure 5E,F). This may suggest that while LASSO effectively refines the lipidomic features for distinguishing ONFH patients from healthy controls, its impact on discriminating between the two osteonecrosis subtypes is less pronounced, indicating a potential overlap in the lipidomic characteristics of TONFH and NONFH.

**Figure 6:**
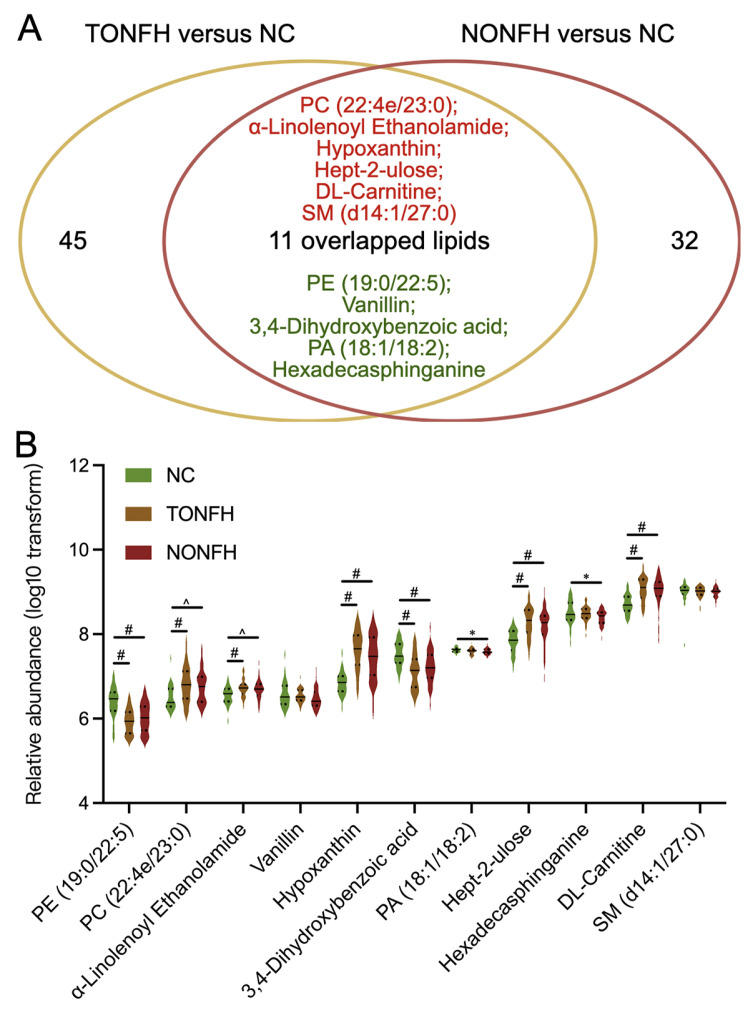
Analysis of relative abundances of the 11 common lipid features. (**A**) Eleven lipid features were identified as overlapping in both TONFH and NONFH compared to the NC; of these, six (indicated in red) were elevated and five (indicated in green) were decreased in the disease conditions. (**B**) Relative abundances of the 11 common lipids across the three groups. Statistical analysis was performed using the Wilcoxon rank-sum test to compare the different groups. * *p* < 0.05, ^ *p* < 0.01, # *p* < 0.001.

Notably, within the above lipid subsets, we identified 11 lipid features that overlapped in both TONFH and NONFH when compared to the NC, which we may consider as common lipid biomarkers for the ONFH pathology (Figure 6A). Furthermore, the abundance changes in these 11 common lipids were consistent in both TONFH and NONFH compared to the NC, with 6 lipids exhibiting increased abundances and 5 lipids showing decreased levels in the disease conditions relative to the control group (Figure 6A,B; Table 2). In addition, several of the 11 identified common lipids demonstrated notable diagnostic potential, with AUC values greater than 0.7 for discriminating between TONFH and the NC, as well as between NONFH and the NC (Table 2). Beyond the 11 lipids common to both conditions, there were an additional 45 lipids and 32 lipids identified by LASSO in TONFH versus the NC and NONFH versus the NC, respectively. These lipid features may be indicative of TONFH-specific and NONFH-specific pathophysiological processes. Notably, several of these subtype-specific markers demonstrated substantial diagnostic potential, with AUC values > 0.7, as shown in Table S1. This suggests that these lipids have potential value as biomarkers for the precise identification and differentiation of ONFH subtypes. Overall, the identification of these 11 common lipids may offer insights into the lipidomic disorders associated with ONFH, irrespective of its etiology. The consistent upregulation and downregulation patterns of the 11 lipids in both TONFH and NONFH suggest that they may play a fundamental role in the pathophysiology of ONFH. The AUC analysis of the ONFH-common lipids and subtype-specific lipids demonstrates their potential application as diagnostic markers and personalized therapeutic intervention targets.

## 4. Discussion

The exploration of the lipidomic profiles in ONFH presents a novel approach for understanding the pathophysiology of ONFH and identifying potential biomarkers for diagnosis and therapeutic targeting. Perturbations in lipid metabolism within bone marrow could potentially have implications for conditions like ONFH, as they may influence the overall metabolic health of the skeletal system and contribute to bone diseases [41,42,43]. Researchers have employed lipidomic analyses to identify the alterations in serum lipid profiles that correlate with glucocorticoid-associated ONFH (GA-ONFH). It has been found that certain lipids, specifically LPE (22:6), CE (14:0), and CE (16:0), have a significant correlation with the early stages of GA-ONFH [44]. Our previous work preliminarily investigated the plasma lipid profiles of patients with ONFH and identified potential lipid biomarkers [12]. Despite these studies, the field lacks an abundance of direct research focused specifically on lipidome analysis in ONFH. In this study, we uncovered significant insights into the lipid metabolism alterations associated with TONFH and NONFH, the two major categories of ONFH. Importantly, the application of UHPLC-MS/MS in metabolome or lipidome research provides a significant advantage due to its sensitivity and resolution, allowing for the comprehensive analysis of metabolites or lipids in biological samples. In addition, we applied the feature selection method of LASSO regression to isolate the most relevant lipid features from the high-dimensional datasets, which enhanced the accuracy of disease characterization and biomarker discovery.

By employing a feature selection approach, we discovered that the lipidome signatures of TONFH and NONFH differ from those of the NC. The identification of 11 common lipid features in both TONFH and NONFH, compared to the NC, is particularly interesting. These lipids exhibit consistent patterns of alteration across both ONFH subtypes, which might suggest a shared pathophysiological mechanism of ONFH at the lipidomic level. The upregulation and downregulation of these lipids in ONFH patients, irrespective of the etiology, points towards a common disruption in lipid metabolism pathways. The accumulation of fat cells in bone marrow, leading to vascular compression and subsequent bone cell death, is a well-recognized phenomenon in ONFH [2,45]. Our study extends this understanding by identifying specific lipid alterations that might be involved in these processes. Furthermore, we explored the diagnostic potential of these lipid biomarkers. The relative abundances of PC (22:4e/23:0), Hypoxanthine, Hept-2-ulose, and DL-Carnitine are found to be increased in both TONFH and NONFH compared to the NC, with their AUC values > 0.7 for both comparisons. Conversely, the relative abundances of PE (19:0/22:5) and 3,4-Dihydroxybenzoic acid are decreased in TONFH and NONFH compared to the NC, also with AUC values greater than 0.7 for both comparisons. These findings indicate that specific changes in lipid and metabolite profiles, characterized by both increases and decreases in certain compounds, are associated with TONFH and NONFH. Mechanically, these lipid alterations may collectively contribute to the pathophysiology of ONFH by disrupting cellular membrane integrity, promoting oxidative stress, impairing energy metabolism, and dysregulating the signaling pathways involved in bone cell survival and function. Additionally, the accumulation of specific lipid species may exacerbate inflammatory responses and vascular dysfunction, further contributing to bone tissue damage and necrosis in ONFH. Further mechanistic studies are warranted to elucidate the precise roles of these lipid biomarkers in ONFH pathogenesis, and to explore their potential as therapeutic targets or diagnostic markers.

Moreover, the additional lipid features identified as subtype-specific markers of TONFH and NONFH underline the subtle differences in the lipidomic profiles of the two subtypes. While TONFH is primarily a consequence of direct trauma disrupting blood flow, NONFH arises from a variety of etiological factors, including prolonged corticosteroid use and excessive alcohol consumption. The relatively different lipidomic signatures of the two subtypes reflect their differing underlying mechanisms. The identification of these subtype-specific lipids not only enhances our understanding of the disease’s heterogeneity, but also improves the possibilities for personalized diagnostic and therapeutic approaches. For instance, the lipid biomarkers specific to NONFH might potentially be used to monitor patients at risk due to prolonged corticosteroid therapy or other predisposing factors. In this study, it was observed that the lipids Glycineamide ribonucleotide, Hypoxanthine, and 4-(1,1-Dimethylpropyl)phenol demonstrate a significant capability for distinguishing TONFH from the NC, with AUC values exceeding 0.9. Similarly, the lipids L-(+)-glutamine, Hypoxanthine, Alpha-GPC, Noramidopyrine, DL-Carnitine, and LPG 18:1 show a notable ability to differentiate NONFH from the NC, with AUC values greater than 0.8. These findings suggest that specific lipid compounds possess strong discriminative power for identifying TONFH and NONFH from the normal controls, underlying their potential as effective biomarkers for TONFH or NONFH.

Furthermore, our study highlights the utility of machine learning techniques in the analysis of complex biological datasets. The high-dimensional nature of lipidomic data poses significant challenges in terms of analysis and interpretation [46,47]. The use of a LASSO regression in our study effectively addressed these challenges, enabling the identification of the most informative lipid features from the entire dataset. This approach not only facilitated a more focused analysis but also reduced the computational burden, exemplifying the potential of machine learning in biomedical research.

However, our study has several limitations that warrant careful consideration. While the sample size was adequate for a preliminary investigation, future studies should include larger, more diverse cohorts to enhance the robustness and generalizability of the findings. Detailed clinical information, such as corticosteroid use duration and dosage for NONFH patients, and the specific type of trauma for TONFH patients, was not systematically collected, which limits the precision of our analysis. Furthermore, the exclusion criteria for the NC group, which relied on both the self-reported medical history and laboratory tests, could still introduce some bias, particularly with undiagnosed conditions affecting lipid metabolism. However, this dual approach minimized potential bias, ensuring that the NC group remained as representative of a healthy population as possible. In addition, while we provided the distribution of the ARCO stages in Table 1, a more detailed exploration of the relationship between lipidomic profiles and disease progression according to the ARCO stages would offer valuable insights. Although the PLS-DA provided meaningful results, the risk of overfitting remains a concern, especially with the small sample size relative to the number of variables analyzed. Future studies should apply cross-validation and independent datasets to confirm the robustness of the lipid biomarkers identified. These limitations highlight the need for follow-up studies with larger datasets, more comprehensive clinical data, and advanced analytical techniques to further elucidate the lipidomic signatures of ONFH and their clinical implications.

In terms of clinical applications, the lipid biomarkers identified in this study hold significant promise for the future diagnosis and therapeutic monitoring of ONFH. Given their strong association with both TONFH and NONFH, these lipid biomarkers could be utilized to develop diagnostic assays, enabling early detection and the differentiation of ONFH subtypes. The identification of specific lipid alterations also suggests potential therapeutic targets, as modulating lipid metabolism pathways may help prevent or mitigate the progression of the disease. For instance, lipid-targeted therapies could be explored to address the underlying metabolic disturbances in ONFH patients, particularly in cases where traditional treatments, such as corticosteroids or surgical interventions, are insufficient. Future research will focus on validating these biomarkers in larger cohorts, optimizing their diagnostic utility, and exploring their therapeutic potential in clinical settings.

## 5. Conclusions

In conclusion, our study conducted a comprehensive lipidomic analysis of NONFH and TONFH, the two major types of ONFH. Utilizing UHPLC–MS/MS and the feature selection method of LASSO regression, we identified the lipidomic signatures specific to TONFH and NONFH. Our findings revealed 11 common lipid biomarkers across both ONFH subtypes. Several of these biomarkers demonstrated significant diagnostic potential, with alterations in lipid profiles, such as increased levels of PC (22:4e/23:0), Hypoxanthine, Hept-2-ulose, and DL-Carnitine, and decreased levels of PE (19:0/22:5) and 3,4-Dihydroxybenzoic acid in TONFH and NONFH compared to the NC. Additionally, we also identified subtype-specific lipid markers, which reflect the unique pathogenic mechanisms underlying TONFH and NONFH. The application of a LASSO regression assisted in the analysis of the high-dimensional lipidomic data and enhanced the accuracy of disease characterization and biomarker discovery. Taken together, our study explored lipidomic profiling and provides clues for personalized diagnostic and therapeutic strategies for ONFH. While this research offers crucial insights into the lipidomic landscape of ONFH and its potential diagnostic markers, further studies are warranted to validate these findings and explore the mechanistic pathways involved. Specifically, future research directions could include mechanistic studies to elucidate the specific roles of the identified lipid biomarkers in ONFH pathogenesis. Additionally, validation in larger cohorts, possibly through multi-center collaborations, is crucial to confirm the diagnostic and prognostic utility of these lipidomic signatures. Overall, future efforts should focus on translating the findings into clinically applicable diagnostic markers and targeted therapeutic interventions for ONFH.

## Figures and Tables

**Table 1:** Clinical information on the participants in this study.

Groups	Number	Age (Years)	Gender	BMI	ARCO Stage	
			Female/Male		I/II	III/IV
NC	33	48.33 ± 8.60	15/18	23.17 ± 2.68	---	---
TONFH	46	49.46 ± 12.38	21/25	23.88 ± 2.73	6/7	18/15
NONFH	46	48.87 ± 14.43	17/29	23.93 ± 2.84	5/8	16/17

**Table 2:** Relative abundances and AUC analysis of the 11 identified common lipids in comparisons of TONFH and NC and of NONFH and NC.

Lipids	Formula	Molecular Weight	Retention Time [min]	Relative Abundances (log10)			Area Under Curves	
				NC	TONFH	NONFH	TONFH/NC	NONFH/NC
PE (19:0/22:5)	C_46_H_82_NO_8_P	807.577	11.412	6.386	5.928 #	6.024 #	0.845	0.775
PC (22:4e/23:0)	C_53_H_100_NO_7_P	893.717	15.368	6.492	6.813 #	6.752 ^	0.738	0.721
α-Linolenoyl Ethanolamide	C_20_H_35_NO_2_	321.267	3.579	6.541	6.720 #	6.689 ^	0.735	0.689
Vanillin	C_8_H_8_O_3_	152.047	0.996	6.572	6.532	6.474	0.512	0.599
Hypoxanthine	C_5_H_4_N_4_O	136.039	0.947	6.834	7.632 #	7.439 #	0.920	0.832
3,4-Dihydroxybenzoic acid	C_7_H_6_O_4_	154.026	0.965	7.524	7.107 #	7.222 #	0.785	0.739
PA (18:1/18:2)	C_39_H_71_O_8_P	698.488	10.837	7.625	7.597	7.589 *	0.605	0.663
Hept-2-ulose	C_7_H_14_O_7_	210.073	0.966	7.836	8.285 #	8.179 #	0.831	0.779
Hexadecasphinganine	C_16_H_35_NO_2_	273.267	0.947	8.506	8.480	8.395 *	0.517	0.619
DL-Carnitine	C_7_H_15_NO_3_	161.105	1.232	8.710	9.065 #	9.020 #	0.836	0.817
SM (d14:1/27:0)	C_46_H_93_N_2_O_6_P	846.682	14.445	8.991	9.001	8.995	0.542	0.567

In the Wilcoxon rank-sum test, comparisons to the NC group revealed * *p* < 0.05, ^ *p* < 0.01, and # *p* < 0.001.

## Data Availability

All the data generated or analyzed during this study are publicly available at https://github.com/jihanwang/LASSO-for-lipidomic.

## Supplementary Materials

The following supporting information can be downloaded at: https://www.mdpi.com/article/10.3390/biomedicines12122827/s1, Table S1: AUC analysis of lipids selected by LASSO for TONFH versus NC and NONFH versus NC.

## References

[1] Rezus E., Tamba B.I., Badescu M.C., Popescu D., Bratoiu I., Rezus C. (2021). Osteonecrosis of the Femoral Head in Patients with Hypercoagulability-From Pathophysiology to Therapeutic Implications. Int. J. Mol. Sci..

[2] Chen Y., Miao Y., Liu K., Xue F., Zhu B., Zhang C., Li G. (2022). Evolutionary Course of the Femoral Head Osteonecrosis: Histopathological—Radiologic Characteristics and Clinical Staging Systems. J. Orthop. Transl..

[3] Hwang Y., Park J., Choi S.H., Kim G. (2011). Traumatic and Non-Traumatic Osteonecrosis in the Femoral Head of a Rabbit Model. Lab. Anim. Res..

[4] Tripathy S.K., Goyal T., Sen R.K. (2015). Management of Femoral Head Osteonecrosis: Current Concepts. Indian J. Orthop..

[5] Li Z., Shao W., Lv X., Wang B., Han L., Gong S., Wang P., Feng Y. (2023). Advances in Experimental Models of Osteonecrosis of the Femoral Head. J. Orthop. Transl..

[6] Wen Z., Li Y., Cai Z., Fan M., Wang J., Ding R., Huang C., Xiao W. (2022). Global Trends and Current Status in Osteonecrosis of the Femoral Head: A Bibliometric Analysis of Publications in the Last 30 Years. Front. Endocrinol..

[7] Zhao D., Zhang F., Wang B., Liu B., Li L., Kim S.Y., Goodman S.B., Hernigou P., Cui Q., Lineaweaver W.C. (2020). Guidelines for Clinical Diagnosis and Treatment of Osteonecrosis of the Femoral Head in Adults (2019 Version). J. Orthop. Transl..

[8] Mont M.A., Salem H.S., Piuzzi N.S., Goodman S.B., Jones L.C. (2020). Nontraumatic Osteonecrosis of the Femoral Head: Where Do We Stand Today? A 5-Year Update. J. Bone Jt. Surg. Am..

[9] Liu N., Zheng C., Wang Q., Huang Z. (2022). Treatment of Non-Traumatic Avascular Necrosis of the Femoral Head (Review). Exp. Ther. Med..

[10] Choi H.R., Steinberg M.E., Cheng E.Y. (2015). Osteonecrosis of the Femoral Head: Diagnosis and Classification Systems. Curr. Rev. Musculoskelet. Med..

[11] Yu X., Zhang S., Zhang B., Dai M. (2022). Relationship of Idiopathic Femoral Head Necrosis with Blood Lipid Metabolism and Coagulation Function: A Propensity Score-Based Analysis. Front. Surg..

[12] Yan Y., Wang J., Huang D., Lv J., Li H., An J., Cui X., Zhao H. (2022). Plasma Lipidomics Analysis Reveals Altered Lipids Signature in Patients with Osteonecrosis of the Femoral Head. Metabolomics.

[13] Rendina-Ruedy E., Rosen C.J. (2020). Lipids in the Bone Marrow: An Evolving Perspective. Cell Metab..

[14] Petek D., Hannouche D., Suva D. (2019). Osteonecrosis of the Femoral Head: Pathophysiology and Current Concepts of Treatment. EFORT Open Rev..

[15] Wang B., Wang H., Li Y., Song L. (2022). Lipid Metabolism within the Bone Micro-Environment Is Closely Associated with Bone Metabolism in Physiological and Pathophysiological Stages. Lipids Heal. Dis..

[16] Moya-Angeler J., Gianakos A.L., Villa J.C., Ni A., Lane J.M. (2015). Current Concepts on Osteonecrosis of the Femoral Head. World J. Orthop..

[17] Kang P., Gao H., Pei F., Shen B., Yang J., Zhou Z. (2010). Effects of an Anticoagulant and a Lipid-Lowering Agent on the Prevention of Steroid-Induced Osteonecrosis in Rabbits. Int. J. Exp. Pathol..

[18] Konarski W., Pobozy T., Konarska K., Sliwczynski A., Kotela I., Hordowicz M., Krakowiak J. (2023). Osteonecrosis Related to Steroid and Alcohol Use-An Update on Pathogenesis. Healthcare.

[19] Birla V., Vaish A., Vaishya R. (2021). Risk Factors and Pathogenesis of Steroid-Induced Osteonecrosis of Femoral Head—A Scoping Review. J. Clin. Orthop. Trauma..

[20] Clish C.B. (2015). Metabolomics: An Emerging but Powerful Tool for Precision Medicine. Cold Spring Harb. Mol. Case Stud..

[21] Fuller H., Zhu Y., Nicholas J., Chatelaine H.A., Drzymalla E.M., Sarvestani A.K., Julian-Serrano S., Tahir U.A., Sinnott-Armstrong N., Raffield L.M. (2023). Metabolomic Epidemiology Offers Insights into Disease Aetiology. Nat. Metab..

[22] Schmidt D.R., Patel R., Kirsch D.G., Lewis C.A., Vander Heiden M.G., Locasale J.W. (2021). Metabolomics in Cancer Research and Emerging Applications in Clinical Oncology. CA Cancer J. Clin..

[23] Ahluwalia K., Ebright B., Chow K., Dave P., Mead A., Poblete R., Louie S.G., Asante I. (2022). Lipidomics in Understanding Pathophysiology and Pharmacologic Effects in Inflammatory Diseases: Considerations for Drug Development. Metabolites.

[24] Zandl-Lang M., Plecko B., Kofeler H. (2023). Lipidomics-Paving the Road towards Better Insight and Precision Medicine in Rare Metabolic Diseases. Int. J. Mol. Sci..

[25] Wang R., Li B., Lam S.M., Shui G. (2020). Integration of Lipidomics and Metabolomics for In-Depth Understanding of Cellular Mechanism and Disease Progression. J. Genet. Genom..

[26] Han X., Gross R.W. (2022). The Foundations and Development of Lipidomics. J. Lipid Res..

[27] Belhaj M.R., Lawler N.G., Hoffman N.J. (2021). Metabolomics and Lipidomics: Expanding the Molecular Landscape of Exercise Biology. Metabolites.

[28] Rampler E., Abiead Y.E., Schoeny H., Rusz M., Hildebrand F., Fitz V., Koellensperger G. (2021). Recurrent Topics in Mass Spectrometry-Based Metabolomics and Lipidomics-Standardization, Coverage, and Throughput. Anal. Chem..

[29] Chappel J.R., Kirkwood-Donelson K.I., Reif D.M., Baker E.S. (2023). From Big Data to Big Insights: Statistical and Bioinformatic Approaches for Exploring the Lipidome. Anal. Bioanal. Chem..

[30] Petrick L.M., Shomron N. (2022). AI/ML-Driven Advances in Untargeted Metabolomics and Exposomics for Biomedical Applications. Cell Rep. Phys. Sci..

[31] Bifarin O.O., Sah S., Gaul D.A., Moore S.G., Chen R., Palaniappan M., Kim J., Matzuk M.M., Fernandez F.M. (2023). Machine Learning Reveals Lipidome Remodeling Dynamics in a Mouse Model of Ovarian Cancer. J. Proteome Res..

[32] Galal A., Talal M., Moustafa A. (2022). Applications of Machine Learning in Metabolomics: Disease Modeling and Classification. Front. Genet..

[33] Pudjihartono N., Fadason T., Kempa-Liehr A.W., O’Sullivan J.M. (2022). A Review of Feature Selection Methods for Machine Learning-Based Disease Risk Prediction. Front. Bioinform..

[34] Saeys Y., Inza I., Larranaga P. (2007). A Review of Feature Selection Techniques in Bioinformatics. Bioinformatics.

[35] Lee I.C.H., Tumanov S., Wong J.W.H., Stocker R., Ho J.W.K. (2023). Integrative Processing of Untargeted Metabolomic and Lipidomic Data Using MultiABLER. iScience.

[36] Acharjee A., Ament Z., West J.A., Stanley E., Griffin J.L. (2016). Integration of Metabolomics, Lipidomics and Clinical Data Using a Machine Learning Method. BMC Bioinform..

[37] Fahy E., Subramaniam S., Murphy R.C., Nishijima M., Raetz C.R., Shimizu T., Spener F., van Meer G., Wakelam M.J., Dennis E.A. (2009). Update of the LIPID MAPS Comprehensive Classification System for Lipids. J. Lipid Res..

[38] Kind T., Okazaki Y., Saito K., Fiehn O. (2014). LipidBlast Templates as Flexible Tools for Creating New In-Silico Tandem Mass Spectral Libraries. Anal. Chem..

[39] Vasquez M.M., Hu C., Roe D.J., Chen Z., Halonen M., Guerra S. (2016). Least Absolute Shrinkage and Selection Operator Type Methods for the Identification of Serum Biomarkers of Overweight and Obesity: Simulation and Application. BMC Med. Res. Methodol..

[40] Li Y., Lu F., Yin Y. (2022). Applying Logistic LASSO Regression for the Diagnosis of Atypical Crohn’s Disease. Sci. Rep..

[41] Tian L., Yu X. (2015). Lipid Metabolism Disorders and Bone Dysfunction--Interrelated and Mutually Regulated (Review). Mol. Med. Rep..

[42] Alekos N.S., Moorer M.C., Riddle R.C. (2020). Dual Effects of Lipid Metabolism on Osteoblast Function. Front. Endocrinol..

[43] Srivastava R.K., Sapra L., Mishra P.K. (2022). Osteometabolism: Metabolic Alterations in Bone Pathologies. Cells.

[44] Wang X.Y., Zhang L.L., Jiang C., Hua B.X., Ji Z.F., Fan W.S., Gong L.J., Zhu L., Wang X.D., Yan Z.Q. (2021). Altered Lipidomic Profiles in Patients with and without Osteonecrosis of the Femoral Head after 1-Month Glucocorticoid Treatment. Clin. Transl. Med..

[45] Wang H., Leng Y., Gong Y. (2018). Bone Marrow Fat and Hematopoiesis. Front. Endocrinol..

[46] Wang J., Han X. (2019). Analytical Challenges of Shotgun Lipidomics at Different Resolution of Measurements. Trends Anal. Chem..

[47] Zullig T., Kofeler H.C. (2021). High Resolution Mass Spectrometry in Lipidomics. Mass. Spectrom. Rev..

