# Supplementary material for: Research on Lipidomic Profiling and Biomarker Identification for Osteonecrosis of the Femoral Head
Source: Biomedicines. 2024 Dec 12;12(12):2827. doi: 10.3390/biomedicines12122827 (PMC11673004; doi:10.3390/biomedicines12122827)
Supplement: Supplementary file 1 [file biomedicines-12-02827-s001.zip › Table S1.pdf]

Table S1. AUC analysis of lipids selected by LASSO for TONFH versus NC and NONFH versus NC

TONFH versus NC		NONFH versus NC	
Lipids	AUC	Lipids	AUC
Glycineamide ribonucleotide	0.9223	L-(+)-glutamine	0.8353
**Hypoxanthine**	0.9196	**Hypoxanthine**	0.832
4-(1,1-Dimethylpropyl)phenol	0.9045	Alpha-GPC	0.8281
N-Acetylvaline	0.8636	Noramidopyrine	0.8182
**PE (19:0/22:5)**	0.8445	**DL-Carnitine**	0.8169
**DL-Carnitine**	0.836	LPG 18:1	0.8169
**Hept-2-ulose**	0.8314	D-617	0.7964
**3,4-Dihydroxybenzoic acid**	0.7852	Serotonin	0.7964
alpha-Glucoheptitol	0.776	**Hept-2-ulose**	0.7787
chlorphenamine	0.7596	**PE (19:0/22:5)**	0.7747
GlcADG (16:0-20:4)	0.749	GM3 d42:2; [M-H]-	0.7589
Ganglioside GM3 (d18:1/24:1(15Z))	0.7451	N-hexadecanoylphytosphingosine	0.7536
**PC (22:4e/23:0)**	0.7378	Cer-NP (t18:1/24:1)	0.7457
**α-Linolenoyl ethanolamide**	0.7345	PC (19:2/18:5)	0.7451
PS(18:0/22:5(7Z,10Z,13Z,16Z,19Z))	0.7266	**3,4-Dihydroxybenzoic acid**	0.7385
LysoPC(18:4(6Z,9Z,12Z,15Z))	0.7227	PE (19:2/19:2)	0.7378
PC (18:0e/18:2)	0.7161	TAG (12:3-16:0-20:4)	0.7339
DG(14:0/18:3(6Z,9Z,12Z)/0:0)	0.7141	Dehydroepiandrosterone sulfate	0.7339
PC (18:2/22:6)	0.7049	Sunitinib	0.7273

quinol sulfate	0.7016	**PC (22:4e/23:0)**	0.7207
TAG (18:0-18:1-22:5)	0.6831	PC (18:3/20:5)	0.7003
PEtOH (27:0-18:1)	0.6825	PEG-4	0.6976
PC (17:1/18:1)	0.6765	**α-Linolenoyl ethanolamide**	0.6891
Paracetamol	0.6765	(2xi)-D-gluco-heptonic acid	0.6792
N-[(15Z)-tetracosenoyl]sphing-4-enine-1-phosphocholine	0.6739	Cer-NDS (d18:0/20:0)	0.6765
HexCer-NS (d18:1/24:1)	0.6726	20-Oxopregn-5-en-3-yl hydrogen sulfate	0.6726
PC (18:1e/22:3)	0.6528	PC (19:2/20:5)	0.6713
CE 16:0	0.6436	TAG (17:0-18:2-20:4)	0.6706
PG (18:1/18:1)	0.6416	PC (20:3/22:6)	0.6686
DAG (18:1/22:5)	0.6403	**PA (18:1/18:2)**	0.6627
TG(18:2(9Z,12Z)/20:4(5Z,8Z,11Z,14Z)/20:4(5Z,8Z,11Z,14Z))[iso3]	0.6252	PC (18:4e/24:4)	0.6594
SM (d14:2/28:0)	0.6252	4alpha-hydroxymethyl-4beta-methylzymosterol	0.6555
PC (22:6e/24:0)	0.6245	PC (22:4e/18:0)	0.6449
PE (18:1e/16:0)	0.6245	PC (18:0/18:2)	0.643
Cer-NS (d18:2/25:0)	0.6232	TG(18:2(9Z,12Z)/18:2(9Z,12Z)/20:1(11Z))[iso3]	0.639
PC (16:1e/24:4)	0.6173	PC (22:3e/20:4)	0.6291
TG(16:1(9Z)/18:2(9Z,12Z)/18:2(9Z,12Z))[iso3]	0.6166	**Hexadecasphinganine**	0.6186
**PA (18:1/18:2)**	0.6054	**Vanillin**	0.5988
TAG (16:1-18:2-22:5)	0.6041	N-(11Z,14Z)-eicosadienoylethanolamine	0.5968
PC (18:0e/20:3)	0.6034	PC (16:0e/16:1)	0.5903
PC (22:4e/16:1)	0.6028	**SM (d14:1/27:0)**	0.5672

Morusinol	0.6021	3-(Hexacosanoyloxy)-4-(trimethylammonio)butanoate	0.5264
GM3 d40:2; [M-H]-	0.6008	1-alpha-linolenoyl-2-arachidoyl-sn-glycerol	0.5132
TAG (18:1-22:5-22:6)	0.5929		
Cer-NS (d18:1/23:0)	0.5777		
1-oleoyl-2-arachidonoyl-sn-glycero-3-phosphate	0.5468		
1,1'-[1,11-Undecanediylbis(oxy)]dibenzene	0.5441		
**SM (d14:1/27:0)**	0.5422		
Cer-NDS (d18:0/23:0)	0.5343		
**Hexadecasphinganine**	0.5165		
TG(18:2(9Z,12Z)/18:2(9Z,12Z)/20:2(11Z,14Z))[iso3]	0.5158		
**Vanillin**	0.5119		
NL5993600	0.5119		
GlcADG (20:2-20:4)	0.5105		
1,3-Dihydroxy-2-propanyl (13Z,16Z)-13,16-docosadienoate	0.5053		
HexCer-NS (d18:2/22:0)	0.5033		

Utilizing LASSO regression on lipidomic profiles, we identified subsets of lipid features with enhanced discriminative capability: 56 lipid features for TONFH versus NC, and 43 for NONFH versus NC. Displayed here are the AUC values for each lipid in the TONFH versus NC and NONFH versus NC comparisons. The 11 lipids highlighted in bold represent the common lipids shared between TONFH versus NC and NONFH versus NC.
